# Rapid Health and Needs assessments after disasters: a systematic review

**DOI:** 10.1186/1471-2458-10-295

**Published:** 2010-06-01

**Authors:** Helena A Korteweg, Irene van Bokhoven, CJ Yzermans, Linda Grievink

**Affiliations:** 1 National Institute for Public Health and the Environment, Postbus 1 3720 BA, Bilthoven; The Netherlands; 2 Netherlands institute for health services research, Postbus 1568 3500 BN, Utrecht; The Netherlands

## Abstract

**Background:**

Publichealth care providers, stakeholders and policy makers request a rapid insight into health status and needs of the affected population after disasters. To our knowledge, there is no standardized rapid assessment tool for European countries. The aim of this article is to describe existing tools used internationally and analyze them for the development of a workable rapid assessment.

**Methods:**

A review was conducted, including original studies concerning a rapid health and/or needs assessment. The studies used were published between 1980 and 2009. The electronic databasesof Medline, Embase, SciSearch and Psychinfo were used.

**Results:**

Thirty-three studies were included for this review. The majority of the studies was of US origin and in most cases related to natural disasters, especially concerning the weather. In eighteen studies an assessment was conducted using a structured questionnaire, eleven studies used registries and four used both methods. Questionnaires were primarily used to asses the health needs, while data records were used to assess the health status of disaster victims.

**Conclusions:**

Methods most commonly used were face to face interviews and data extracted from existing registries. Ideally, a rapid assessment tool is needed which does not add to the burden of disaster victims. In this perspective, the use of existing medical registries in combination with a brief questionnaire in the aftermath of disasters is the most promising. Since there is an increasing need for such a tool this approach needs further examination.

## Background

### Importance of rapid assessments

When disaster strikes it is important to realize that apart from acute health problems that will be addressed by the emergency departments many other problems are likely to occur [1]. Homes may be damaged, sometimes resulting in displacement of the population. Survivors might develop diseases or have other health problems as a consequence of the disaster. These problems may result in health related needs like medical treatment and medication use. Since a disaster might have direct consequences for public health care a clear overview of these health needs is important. Therefore rapid assessment methods are needed to collect reliable, objective information that is immediately required for decision making in the recovery phase of the event. Health care agencies, stakeholders and policy makers will request a rapid insight into health status to take care of the needs of the affected population [2]. With this collected information about health status and needs, public health interventions can be prioritized. Rapid assessment tools are also important to guide the emergency efforts in the affected area [3]. For example, public health interventions and emergency efforts may include improvements of access to medical care, financial support and restoration of damaged houses.

Since health needs can rapidly change [2] after the acute phase and a quick insight into common health problems is important to preserve adequate health care, this article focuses on assessment methods which can be applied in the first two weeks after a disaster. This is also important because collection of possible exposure data, such as the extent of involvement or the use of protection measures, is the most reliable in the first two weeks after an event (to prevent recall bias). Furthermore, we assume that a rapid assessment can provide information that can be necessary in case the need for the regular local health and medical systems is unknown or if these systems are overloaded or disrupted due to the disaster. After all, if the regular local health care is operative no information is needed for collective health care.

### History and background research

In the 1980's the development of rapid assessment tools started in the United States. In 1999, this resulted in the Rapid Health Assessment Protocols for Emergencies developed by the World Health Organization (WHO) [5]. These protocols were developed to determine the immediate and potential health impact of a broad range of emergencies, such as epidemics, natural disasters and chemical emergencies [5]. The Centers for Disease Control and Prevention (CDC) in the United States also developed a rapid assessment tool to measure practical needs, health status and health needs [6,7]. In the Netherlands health assessments after disasters so far focused on other methods, such as surveys with a time frame starting from three weeks to a few years post-disaster [8] and surveillance studies that were operational within a few months [9,10]. No standardized rapid assessment tool is available for the Dutch population and, to our knowledge, for other European countries to assess expected health needs. This can result in failing to meet the actual needs after a disaster. Because emergencies are often complex, it is important to collect information systematically using a standardized tool [5].

### Objectives and primary goal

Which type of rapid assessment tools are developed and used internationally is the main question that forms the basis of this article, in which is examined which aspects of assessments may influence the rapidness such as preparation and procedure of assessment. The primary goal of this article is to describe and analyze these existing aspects which will contribute to the development of a useful rapid assessment tool. With this review we will show what is internationally known in the literature and to show any possible gaps of information in the literature. Ideally a tool is needed which does not add to the burden of disaster victims. This is an important consideration when collecting health information about disaster victims. We will discuss some aspects that might add to or relieve this burden and view and compare the most commonly used rapid assessments in this light. This article focuses on assessment of health status and needs; however, when disaster strikes other consequences such as exposure that can influence the health of affected people needs to be considered and/or incorporated to minimize the burden of survivors and to restore their collective control [1].

## Methods

### Search strategy

To identify the existing assessment methods in literature we conducted a systematic review. We started our search by defining search terms, which were categorized into four categories (table 1).

**Table 1:** Search terms

A. Disaster-related	disaster, crisis*, (mass) emergency*, life event*, traumatic event*, environmental exposure, calamity*, mass accident
**B. Methods**	assessment*, method*, protocol*, concept*, system*, procedure*, design, survey, record

**C1. Health related**	-(immediate/pre-existing) health problems, health status, health conditions -stress, distress, concerns, worries, anxieties, psychotrauma -somatic symptoms/complaints, physical symptoms/complaints, diseases, illness, casualties and fatalities/injured and wounded, dead, death rates, morbidity

**C2. Needs**	- (immediate) health needs, care needs, medical needs, medical services, medicine needs, aftercare needs, psychosocial needs -practical needs, logistic needs, communication needs, accommodation needs, food needs, financial needs, information needs

* An asterisk was placed at the end of some words to search for all terms that begin with that word

Five electronic databases were searched: MEDLINE (NLM); EMBASE (2008 Elsevier B.V.); SciSearch (The Thompson Corporation); PsycINFO (AM. PSYCH. ASSN. 2007) and Social SciSearch (The Thompson Corporation). The categories were combined as follows: **A **AND **B **AND (**C1 **OR **C2)**. We included scientific articles and books. The search was extended by examining references of the reviewed articles. In addition to literature in the English language, we included literature in Dutch and German. As mentioned in the background, the development of rapid assessment tools started in the 1980's. Therefore, we reviewed literature that was published between 1980 and May 2009.

All titles and abstracts of the studies identified by the search in the electronic databases were screened by one of the authors to evaluate whether the inclusion criteria were met (H.K.). A selection of the abstracts was screened in a similar fashion by a second author (I. v. B.) to check whether the inclusion criteria were reproducible by a colleague researcher. Full text versions of all selected potentially relevant articles were judged (H.K.) against the inclusion criteria. In case of doubt, a second (I.v.B.) and or third (L.G.) author was asked to evaluate these articles.

### Inclusion criteria

The inclusion criteria were as follows:

1) Disaster criterion

Studies in the context of man-made (e.g. explosions, aircraft disasters) or natural disasters (e.g. hurricanes, earthquakes) were included. In this study, a disaster is defined as a collective stressful experience with a sudden onset which causes disruption of a community. Studies about individual traumas, war, and drought conditions such as malnutrition did not comply with this definition.

2) *Outcome criterion*

Studies in which health status and/or health needs of disaster victims are the measured topics (table 2) were included. Health status includes the actual immediate health problems and pre-existing health problems. This provides information to assess the immediate health needs of the affected groups. The focus of needs is on medical, housing and logistical issues. We included articles in which the health status and/or needs were actually measured. We excluded the studies if the assessed topics were not described.

**Table 2:** Topics measured of the articles included

Demographic information	Health status	Health needs	Practical needs & status
- gender & age - household composition - employment - educational status - ethnicity	- current physical status - physical status pre-disaster - acute conditions due to the event (injuries) - chronic conditions - illnesses	- medication needs - medical needs acute & pre-disaster	- residence status(damage/inhabitable) - electricity - water & food - communication - transport - utilities & service needs (e.g. child care, religion, schools)

3) Specific health status criterion

Studies that were included report the physical health status like injuries and disaster-related diseases. Studies focusing exclusively on mortality or mental health disorders (in particular PTSD) of disaster victims were excluded. Mental health disorders are excluded, because they cannot be established within two weeks, our definition of a rapid assessment, after the disaster [11].

4) Population criterion

Adultsand children who were directly exposed to a man-made or natural disaster were included. Relief workers were included; except if relief workers themselves were not directly exposed to the disaster.

5) Rapid criterion

Studies in which the assessment started in the first two weeks after a disaster were included. Our definition of rapidness in this review is two weeks, since needs change rapidly over time. If the assessment was not performed within this period, we included studies if the assessed method could have been used within this period. In order to determine whether this was possible we addressed the following questions:

• Was the method or instrument (e.g. interview, surveillance) described?

• Was the description available on how the assessment was conducted? (e.g. face to face, an interview by telephone or self-reported questionnaires)

• Is the moment (time after disaster) of measurement and duration of the assessment described?

A study was excluded when relevant information was absent to answer one of these questions. When it was obvious that the duration of the assessment was too time-consuming the study was excluded. We did not use a clear cut-off for the duration of a rapid assessment, but when the duration was several months we considered this too time-consuming.

### Data processing

The articles were grouped by the method of data collection. In this article we examined which rapid assessment tools are most commonly used. For each paper is described which aspects of assessments might influence the rapidness of an assessment. We distinguished the following aspects which possibly influence the rapidness of assessments: 1. Preparation of assessment, for example how a questionnaire is prepared (e.g. new checklist developed or checklist translated) 2. Time of assessment after disaster 3. Details of method of data collection, for example how a questionnaire is administrated or how data is registered 4. Level of assessment (e.g. at individual or group level) 5. Source of information, for example who registered data and 6. Location of assessment. We will describe these aspects to be able to make well considered choices concerning the development of a useful rapid assessment tool. Furthermore we will search for a method which is the least demanding for affected people. Therefore we will discuss and compare the results in the light of a possible burden of survivors.

## Results

**Figure 1:**
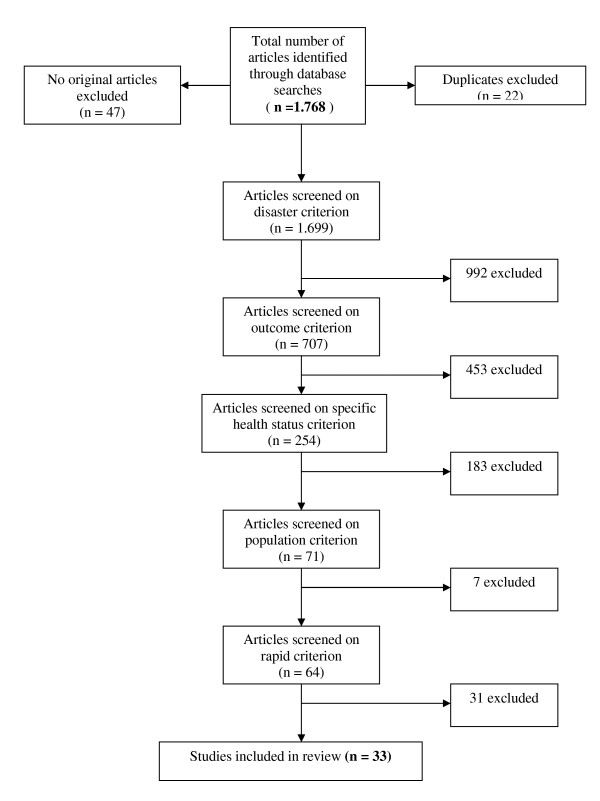
**Flow diagram of the reviewing process**.

The search resulted in 1.768 titles, excluding 47 titles which were no original articles and 22 titles because they were double in the search (Figure 1). Out of these 1.768 titles, 31 articles were excluded from this review because of the definition we used for rapidness. 33 articles were accepted for this review using our inclusion criteria. The accepted articles were divided into two types of methods: structured questionnaires (n = 18) and registries (n = 11). Registries are systems in which routinely collected health information is registered. Four studies used both questionnaires and registries to assess health status and/or needs.

The topics measured were divided in four categories: demographics, health needs, health status and practical needs (see table 2). Structured questionnaires were primarily used to assess the health needs, while registries were used to assess the health status of disaster victims. All twenty-two studies which used interviews covered three or four topics (22 demographics; 19 health needs; 22 health status; 18 practical needs). Most of the studies which used registries (13/14) assessed data about health status such as injuries and illnesses.

In 'Additional file 1' and 'Additional file 2' for each study the type of disaster and the country in which the disaster took place is described, among other things. Disaster type was reported in order to examine whether there was an association with the type of assessment used. The majority of assessments (31/33) were performed after natural disasters. The other two studies were reported after man-made disasters. No association was found between type of disaster and type of assessment. Of the 38 assessments identified for this review, thirteen were assessed after Hurricane Katrina (hundreds of thousands evacuees, 1836 fatalities). These assessments were at thirteen different places and used different information sources such as disaster victims themselves, registries from military hospitals and registries from general hospitals.

### Time of assessment

Data collection with the use of registries had on average a longer time-frame than data collection with the use of questionnaires. In table 3 the start of measurement combined with duration of data collection is summarized. Details about start time and duration can be observed in 'Additional files 1 & 2. Most measurements (n = 29) started within the first two weeks post-disaster. Of these, 17 measurements started in the first two weeks had a duration which was shorter than one week. Most of these studies (14/17) used questionnaires, in the other three studies registries were used. Ten measurements had a duration which lasted longer than two weeks; these assessments were performed with the use of registries.

**Table 3:** Time-frame post-disaster in which the data was collected

Start assessment post-disaster	Duration of data collection			
	
	< 1 week	1-2 weeks	> 2 weeks	Total**
≤ 2 weeks	17	2	10	29

> 2 weeks*	5	4		9

* = possibly rapid (studies which could have been assessed within 2 weeks)

** 33 studies were included, four studies [14,15,28,29] used both types of method (questionnaire & registry) and were therefore included twice in the review separately for both assessments. In one [29] of these four studies even third assessments that each had a different time-frame were described. The total number of assessments was 38.

### Structured questionnaires

Twenty-two studies used a structured questionnaire as assessment method (Additional file 1).

#### Preparation of the questionnaire

Development and preparation of a questionnaire is time consuming. For rapid assessments time is crucial, therefore we examined which aspects of preparation were present in the studies. The following aspects of preparation were distinguished: 1. Modification of a checklist 2. Translation of a checklist and 3. Design of a new checklist. In most studies (15/22) only one of these aspects of preparation was present. In three of these 15 studies [8,20,27] multiple aspects of preparation were present which can be too time-consuming for rapid assessment (3/22). Nine studies used a modified checklist; eight of these studies used templates previously used by the CDC. Four studies [8,12,15,26] developed a new checklist after the disaster. In seven studies it was unclear whether an existing, modified or new checklist was used. However all of these seven studies were also performed with assistance of the CDC. Since eight studies performed by the CDC used a modified checklist, we assume these nine studies also used a modified checklist previously used by the CDC. In five [8,17,20,24,27] of the twenty-two studies a questionnaire was translated, two [20,27] of these questionnaires were also modified to the specific disaster situation and one of them was also newly designed [8].

#### Method of data collection

The way an assessment is conducted influences how rapid the data can be collected. The majority (20/22) of the questionnaires were administered face to face by means of an interview. One study [15] used the telephone to collect information and in another study [8] the questions were self-reported by disaster victims. In most of the studies (17/22) in which face to face interviews were used it was unclear whether they used paper or digital versions of the questionnaire. None of the publications described their choice of data collection method.

#### Assessment level

Rapidness of data collection can also be affected by the level at which an assessment is conducted. Questioning all disaster victims at individual level, for example, is more time-consuming than questioning at group level. In most of the studies (19/22) in which a questionnaire was administered the head or a representative of the household was interviewed. In addition to assessment level, the total number of included survivors (respondents) affects the rapidness of data collection. Twelve (12/22) of the studies had between 100 and 300 respondents, six (6/22) studies had between 300 and 500 respondents and three (3/22) studies had more then 1000 respondents. The individually administered questionnaire had the most respondents (N = 3.792). In this study the researchers' goal was to include all survivors of the disaster [8].

#### Location of assessment

In all twenty-two studies the disaster caused relocation of part of the affected population. In twenty (20/22) of these studies which used structured questionnaires the researchers made a visit to the residence of the disaster victims. Seven (7/22) studies were performed in evacuee centres, thirteen (13/22) in respondents own homes. In one study (1/22) the disaster victims visited a research centre which was especially built [8]. Finally, in one study [15] there was no direct contact between the interviewer and the disaster victims, because interviews were held by telephone.

### Registries

Sixteen studies used registries to collect data about health status and needs (Additional file 2). Seven [25,32,34-37,39] of these studies used more than one type of registry. In one of these seven studies three different registration systems were used. In total twenty-four different registries were used.

#### Method of data collection

We found two different data collection methods. In sixteen of these registries data was abstracted from existing registrations. Data was assigned into categories after the information was collected. In eight registries data was actively recorded on a specific standard disaster form. With this method, information was directly assigned to pre-chosen categories. In two [32,35] of these eight registries health status was directly entered in a computerized disease registration system. In four studies [36-38,41] data was entered on a paper form. In the last two studies [29,42] it was unknown whether data was entered on a paper or a digital form. The majority of the sixteen studies that used regular registration systems did not mention whether these were electronic databases or paper hard copies.

#### Source information

In most of the studies (15/16) data was registered by medical personnel independent of type of registration. In eight studies (8/15) registration was a standard procedure during medical treatment of patients. Seven of these (7/8) studies used Emergency Department (ED) logs reported by ED personnel. In eight studies (8/15) data was collected by medical personnel for purpose of injury and illness surveillance. In five of these (5/8) studies medical staff completed a disaster form for each disaster-patient visit. In two of these studies (2/8) a surveillance team itself completed the disaster form. In one study (1/8) it was unknown who completed the disaster from. Other medical records that were used were pharmacy records (1/15) [35] records from a military hospital registration (1/15) [34] and records from a temporary medical service system (1/15) [25]. In four studies [32,35-37] both existing medical records and a surveillance form were used. In the sixteenth study the Red Cross household registration was used [14].

#### Assessment level

In fifteen of the sixteen studies which used registries the data was collected at individual level. These findings were collected at individual level but reported at population level. In one study [14] the research level was a total household. In this study the Red Cross household registration was used to provide household demographic information about the health needs of the households.

To get insight into the number of persons who can be part of this type of research (use of existing registrations) we examined the number of participants. Two studies had between 200 and 500 participants, five studies had between 1.000 and 6.000 participants, four studies had between 10.000 and 25.000 participants and four studies had between 50.000 and 125.000 participants.

### Comparison between the use of structured questionnaires and registries

We examined the association between level of assessment with the type of assessment used (table 4). We found that type of assessment is associated with level of assessment. Structured questionnaires were mostly (20/22) assessed at household level and data from registries was mostly assessed at individual level.

**Table 4:** Type of assessment linked with level of assessment

Type of assessment	Level of assessment	
	
	Household	Individual
Structured questionnaire	20	2

Registries	1	15

Finally we observed that with the use of existing registries comparison of health status in a disaster situation with a non-disaster situation is possible. Six studies compared data of registries with data from a reference group or reference period. In one of these studies [25] data in the disaster area was compared with similar data from a normal registration system in a non-disaster area during the same period post-disaster. Five studies compared data of registries within their own data (reviewed ED logs). One of these studies [28] compared data with the same period one year earlier, one compared data from eight months pre-disaster with one month post-disaster [33] and one study compared the five days post-disaster period with the 20 days pre-disaster period [32]. Two studies [29,40] compared data of registries over one-week post-disaster within their own data over one-week pre-disaster. None of the data assessed using structured questionnaires was compared with data assessed pre-disaster or in a non-disaster situation.

## Discussion and Conclusions

This review examined which rapid assessment tools are developed and used internationally. A distinction was found between the use of structured questionnaires and the use of registries for rapid assessment of health and needs after disasters. Methods most commonly used were face to face interviews and data extracted from existing registries. Registration systems were used principally to assess health status of survivors while interviews were used primarily to assess health needs. Furthermore, we observed many aspects which influence the rapidness of assessment. Preparation and method of data collection seem to be the most important aspects. Face to face interviews with the use of existing questionnaires was the most rapid manner to collect information about health needs of survivors.

### Influence of the observed aspects on the rapidness of assessments

When performing rapid assessments it is important that the time-frame of assessment is short, because information gathering shortly after a disaster is an important step in assessing the needs of affected people [43]. Several factors concerning these assessments have a potential influence on this time-frame. First of all the time of measurement after the disaster in combination with the duration of the assessment and the time it takes to process the results. The combination of these aspects determines in which time-frame one can share the collected information with health care agencies and policy makers. So it seems to be important for these agencies to know how soon after the disaster the collected data must be available in order to choose the most appropriate method. For example, when using data from registries it is important to know over which period post- and predisaster information is available. When information becomes annually available this registration system obviously is not useful for rapid assessment. It also important to know how much time it will take to extract the data from the existing registries.

Using the rapid criterion we learned about some factors which determine the rapidness of an assessment tool. With this criterion we included studies in which the assessment was or could have been performed in the first two weeks after a disaster.

First we examined the reasons why studies (n = 9) did not start in the first two weeks after a disaster but could have done so within this period:

a) Reason of convenience to collect data later. In one study survey data was collected at the same time a charitable institution distributed monetary aid in emergency centres (> two weeks post-disaster) [18]. In two studies returned evacuees were interviewed who came back home after more than two weeks post-disaster [15].

b) Reason of ethical regulation of study approval by a medical ethical committee. Due to ethical regulation survivors had to receive written information about the study [8].

c) Reasons of lack of preparation. In five studies questionnaires had to be designed and or translated first [8,12,15,22,26].

Secondly, we examined why health and needs assessments (31/64) were excluded from this review because of the definition we used for rapidness. We examined these assessments in order to get insight into which aspects and how these aspects influence the rapidness of assessment. In twelve studies (12/31) it was clear the method used was too time-consuming. For example, the length of the questionnaire was too long (took one and a half hour to complete) [44]. Other studies, for example performed medical examinations [46-49] collected blood and urine [45] to assess the health status or performed vaccination measures [50] in addition to interviews. These time-consuming extra health measures are not favourable for rapid assessments. Most of these articles (19/31) were excluded because relevant information was absent to decide if the assessment was rapid or possible within 2 weeks post-disaster.

From the studies that were included in the review we observed several preparation aspects which influence how rapid a questionnaire can be administered. It is of vital importance that relevant organizations have existing validated questionnaires at their disposal. This review showed that in most studies existing questionnaires had to be modified to the specific disaster situation. This indicates that modifying a questionnaire is possible and often necessary for rapid assessment. A second aspect of preparation is translation of the questionnaire, which is important whenever foreign speaking people are involved in the disaster. It saves time to translate questionnaires at forehand in foreign languages which are common in a certain area. We assumed that if multiple aspects of preparation were present this possibly can be too time-consuming for rapid assessment. However, in two studies modification, translation and conducting the questionnaire was possible within the first two weeks [20,27]. The data reported by Bayleyegn [20] was collected with help of a sufficient number of interviewers who were health professionals. This allowed completion of the survey in relative short time. An American assessment team in the survey of Daley [27] recruited Turkish volunteers who helped review the Turkish version of the questionnaire after an earthquake disaster in Turkey. This Turkish version of the questionnaire already existed and only the modifications needed to be translated.

Also the method of assessment used influences the rapidness of assessment. Most studies used face to face interviews, which appeared to be a quick method, because time can be saved as researchers can immediately collect the results. The responders can not choose their time to fill in the questionnaire. A telephone interview also gives direct access to answers. It is important in rapid assessments that the researcher decides when the questionnaires are conducted and not the interviewee. In combination with the use of a computer that directly records the answers, the rapidness of assessment will be increased. Considering the two registration methods different advantages were observed. Most of these studies used data from existing registrations, which can save time because researchers do not actively need to collect data; they only have to abstract data and assign into categories. On the other hand, when data are actively recorded time can be saved because data are directly assigned into pre-chosen categories. An ideal situation would be when data are directly recorded into a computer on a specific disaster form.

Collection of data at a central location with direct access to completed questionnaires is favourable. Therefore the choice of location also contributes to the rapidness of conducting interviews. Location also influences travelling time of researchers. Assessment at a central place (e.g. an evacuation or research centre) is less-time consuming then interviewing people in their homes where researchers have to go to different locations.

The level at which information is collected (e.g. at individual or group level) is also an aspect that influences the time it takes to perform an assessment. The modified cluster sampling method of the WHO [51] provides health information at household level. This is the most commonly used sampling method when using structured questionnaires to conduct rapid assessment of needs after natural disasters. This method is in particular useful with a geographically dispersed population. Cluster sampling divides the population into groups, or clusters. A number of clusters are selected randomly to represent the evacuated population or an entire affected community. It is a representative method [20] and is less time-consuming than interviewing all disaster victims. Furthermore this sampling technique requires fewer resources. Data collected using registries is mostly collected at an individual level. When data is abstracted from existing registries data from thousands of persons can be collected in a relative short time.

Considering source of information using registries it appeared that data was mostly registered by medical personnel. Half of the studies collected data especially for the purpose of health assessment after disaster, in the other studies registration was a standard procedure during medical consultation. When registration is a standard procedure, medical personnel do not need to invest extra time in data collection.

In short, we conclude that preparation of questionnaires and research, time of measurement, choice of research location, the method of assessment, level of assessment and extent of the survey are all important factors which may influence the rapidness of assessment.

### Aspects of rapid assessments which might add to or relieve the burden of disaster victims

An important topic that needs attention when collecting data after disasters is the burden of disaster victims. There is a growing recognition that collecting health information from the survivors should not aggravate their health. Ideally, a rapid assessment is needed which is the least demanding for disaster victims. After all, the primary goal of health assessments is to collect information that supports the care of disaster survivors. Therefore we will discuss the results in this light. To our knowledge no literature exists that explicitly studies which aspects may influence the burden of survivors. We assume that the lower the number of survivors included in an assessment and the fewer the asked tasks for survivors, the lower the burden for the group as a whole or for the individual. With this assumption the following aspects of rapid assessments were observed which may add to or relieve the burden.

1) The use of data from existing registration systems. We observed that almost all data was routinely collected by medical personnel independent of type of registration. This way affected people were not additionally burdened. Viewing registrations in this perspective, Stalling argued et.al [52] that researchers can intrude into people's lives at the worst possible moments. Disaster researchers commonly justify intrusion to collect knowledge with the aim to reduce suffering and improve response in future disasters. Yet the cost of this gain of knowledge might be disproportionally by subjects. This indication supports the use of existing registrations for health assessment. When data is collected with use of registrations no direct contact with survivors is needed. This means that researchers do not have to intrude into the lives of survivors.

2) Location of assessment may influence the effort it takes for survivors to participate in a survey. Interviewing affected people in their own homes or in evacuation centres, where they were located, might be less demanding than interviewing people outside their residence. Nevertheless, an advantage of a central place to the survivors is that they have the possibility to meet neighbours and friends in particular after evacuation. This social component has been observed in the Netherlands; survivors were very positive about meeting friends and neighbours in a research centre. Personal contact with other survivors might contribute to restore individual well-being.

3) Taking a representative sample of all disaster survivors when using questionnaires to collect information; fewer survivors need to be burdened. However, survivors might feel excluded from participation in the study, in particular if exposure is measured and survivors are worried about exposure. For each specific disaster situation the pros and cons need to be considered. In general for large scale disasters we recommend to use a sample size that has a reasonable margin of error.

4) The magnitude of research depends also on, for example, the length of a questionnaire; ideally the magnitude of research should be minimized in the rapid phase. Only information that is immediately necessary, that needs to be collected quickly to minimize bias, or that might get otherwise lost, should only be considered in this phase.

An important question in this perspective is to what extent do assessments contribute to the survivors feeling of control over oneselves? Did it help them to relieve the impact of the disaster? After the acute phase in which acute care is given, other health aspects might not have first priority because they might be primarily occupied with surviving. However, assessments might positively contribute to the feeling of control in survivors because attention is paid to their needs. Anecdotally evidence exists in the Netherlands and from the CDC (Alden Henderson, personal communication) that disaster survivors experience it positively when their needs are addressed by a face to face interview. Disaster victims often evaluated this as positive in that the government is paying attention to their needs. These questions deserve further research. We recommend to interview survivors about this topic after approximately 3-6 months post-disaster in focus groups. Results from this research can serve as input for the development of the rapid assessment tool.

### Literature and limitations

Our search strategy resulted in 1.768 articles, more than half of these studies were excluded because of our definition of disaster (n = 992). To minimize missing relevant articles we had chosen a broad range of keywords related to disaster. Search terms such as "traumatic event" and "life event" appeared to be too general. As a result, many studies were excluded because they concerned individual events.

In this review we examined why health and needs assessments were excluded from this review because of the criterion "rapid". The most important reason was that relevant information was missing in order to decide whether a rapid assessment was possible. Often information was lacking about the period in which the assessment took place and about its duration. Some articles did not describe information on how a questionnaire was conducted. If we did include all health and needs assessments, we probably would not have drawn different conclusions. About half of the studies which are excluded because of the 'rapid' criterion used questionnaires and about half of these studies used registries. Most of these studies also performed assessments with use of face to face interviews and existing registries.

Although rapid assessment tools were developed for a broad range of emergencies (WHO & CDC) this review showed that rapid assessments were particularly conducted after natural disasters. The studies were in particular performed after hurricanes with at least tens of thousands people involved. Although disasters of this scale hardly ever occur in Europe, we consider these studies to be very informative for the development of a rapid assessment tool, because we are principally interested in the method of assessment. These assessments could be used after different types of disasters and mass emergencies because every event has direct consequences for the public health care.

Publication bias could have affected our results; possibly many conducted rapid assessments were not published in peer-reviewed journals. Publishing articles might not have priority because the primary goal of health and needs assessments is to collect data to support the care and needs of the survivors. This primary goal also indicates that health and needs assessments should not be demanding for disaster victims. Surprisingly, the burden of disaster victims seems no topic of discussion in the literature describing health assessments after disasters.

Furthermore, in all reviewed articles information about processing time of the results is missing. This makes it difficult to estimate the total duration of the assessments. Because of this, we cannot draw conclusions about when information is communicated to policy makers and health care providers. It is important that data is analyzed quickly, so that results can be available as soon as possible [5]. In most of the studies it is also unclear whether paper or digital versions of questionnaires were used. Although we assume digital versions of questionnaires increase the rapidness of an assessment, we cannot draw conclusions about the influence of paper or digital versions of a questionnaire on the rapidness of an assessment. Also, none of the studies described their choice of data collection method. Therefore, we cannot discuss the considerations researchers make about their choice of data collection to make a rapid assessment possible. It was also unclear why assessments sometimes were performed in evacuee camps and sometimes in the most affected communities at people's own homes. We recommend author's of future papers to describe their methods more extensively on how and why a certain method is used and developed. In general we observed that a lot of NGO's who perform rapid assessments after disaster do not publish their findings. This is understandable because their primary goal is the immediate relief of the needs of disaster survivors. Nevertheless we recommend NGO's to publish their findings after their primary goals are reached, because we consider it very important to internationally share the lessons learned.

From the ten studies performed after hurricane Katrina we learned that after a disaster of such enormous scale, several assessments in evacuation camps and in peoples own homes were necessary to get a complete view of health status and needs of affected people. It is possible that more rapid assessments were performed at different locations after other disasters also, but that these were not published.

Finally, we found that some studies compared data of registries with data from a reference group or reference period. In contrast, none of the studies that used structured questionnaires compared their results with data from a reference group or reference period. Comparison with the same kind of data assessed pre-disaster is often not possible.

## Conclusion & Recommendations

In conclusion, this review shows that questionnaires were primarily used to assess health needs and registries to assess health status. Questionnaires were also frequently used to assess health status, but registries were rarely used to assess health needs. In practice, questionnaires are sufficient to assess health status and needs. However, to minimize the possible burden of survivors we prefer the use of registries to assess health status and needs if possible. The use of existing registries also makes it possible to routinely collect information. Another advantage of the use of existing registries is the possibility to compare the health status in a disaster situation with a non-disaster situation. Comparison of data from registries provides longitudinally information about possible increase of illnesses, injuries or hospital visits due to the disaster. In general, the use of reference data provides insight into the actual need for health care and whether this need is different or more extensive than the needs regular health system normally deals with. This may provide direction for public health interventions. We also found that with the use of registries a large number of participants can be included in a survey, showing that registries can easily deal with a large amount of information. Nevertheless it is important to realise that it is not possible to internationally develop a standardized registration system, because the possibilities in each country are different. For example European countries have different types of health registries and different privacy rules to use the data for health research purposes. Furthermore, this review showed that the most commonly used registries are hospital registration systems. When deriving the health status from hospital registration systems only the most severe conditions will be found. In the Netherlands, we have experience with an ongoing surveillance program of health problems registered by general practitioners after a disaster [9,10]. If the disaster did not disrupt the normal health structure, usually people will visit their general practitioner in stead of a hospital. To prevent lack of information we recommend assessing also information from registries of general practitioners apart from hospital registrations. To use these medical registries rapidly, preparation is essential.

Health needs can be derived from health status, for example which medications are needed. But not all needs can be established with registries, for example access to food and water and personal health needs other than medical necessities are important to consider. To assess this kind of information a supplementary questionnaire is necessary. A questionnaire is also necessary in case access to existing registrations is not rapidly possible.

Summarizing we recommend the use of registries in combination with a brief questionnaire for rapid assessment of health status and health needs. Development of this questionnaire needs to be carefully prepared in a non-disaster situation. First the content needs to be established and should be combined with (personal) exposure assessment as much as possible [53]. Second, decisions should be made about translations of the questionnaire to prepare for possible population groups. Third, it is important that the researcher collects data directly; telephone or face to face interviews are for this reason recommended for rapid assessment. Furthermore, the method, use of questionnaire or existing registration, should be operational within two weeks post-disaster. Finally we must be aware that if a large scale disaster with tens or hundreds of thousand evacuees strikes, several assessments in the first weeks post-disaster might be necessary.

Overall, it is important that the rapid assessment tool can be applied after all types of disaster when the regular health system is disrupted or overloaded. In general special attention should be directed to vulnerable groups like people with pre-existing health conditions, pregnant women and vulnerable elderly. This is important because these sensitive subpopulations concern people with unique health needs. For example, it can be more difficult for them to evacuate after a disaster or to obtain access to the medical services they need [54,55]. Beyond the issues of measurement we recommend the development of a standardized questionnaire which can be used internationally. This makes it possible to compare the data that is unambiguous. Preferably one questionnaire will be developed with different modules. This modules are sets of questions that can be modified to the specificity of the disaster situation such as type of disaster and country. A basic set of questions can be developed for each disaster situation, such as disaster involvement (e.g. passenger or citizen) and the experiences and losses due to the disaster. This standardized questionnaire makes it possible to internationally compare the data that is unambiguous. This review summarizes the existing questionnaires which can serve as a starting point to develop a standardized questionnaire.

## Competing interests

The authors declare that they have no competing interests.

## Authors' contributions

HAK selected the literature with assistance of IvB and LG. HAK interpreted and analyzed the literature and also drafted the manuscript. All authors' critically reviewed and approved draft versions and the final manuscript.

## Pre-publication history

The pre-publication history for this paper can be accessed here:

http://www.biomedcentral.com/1471-2458/10/295/prepub

## Supplementary Material

Additional file 1 **Assessments conducted with use of questionnaires**. Overview and characteristics of included articles. Click here for file

Additional file 2 **Assessments conducted with use of registries**. Overview and characteristics of included articles. Click here for file
