# Supplementary material for: Rapid Health and Needs assessments after disasters: a systematic review
Source: BMC Public Health. 2010 Jun 1;10:295. doi: 10.1186/1471-2458-10-295 (PMC2889870; doi:10.1186/1471-2458-10-295)
Supplement: Additional file 1 — Assessments conducted with use of questionnaires. Overview and characteristics of included articles. [file 1471-2458-10-295-S1.DOC]

**Additional file 1. Assessments conducted with use of questionnaires**

| **First Author**  &  Publication Year | **Type of Disaster** Country | **Preparation* questionnaire** | **Method of collecting results**** interviewee# | **N**  **= Respondents** | **population^** | **Time**  **POst disaster** | **Duration** | **SELECTION** |
| --- | --- | --- | --- | --- | --- | --- | --- | --- |
| **1. Greenough 2008** | Hurricane  U.S.A. | new | Face to face (individual) | 499 | Evacuees | 16 days | 10 days | Cluster  sampling |
| **2. Ghosh**  **2007** | Hurricane  U.S.A. | modified  (CDC) | Face to face  (household) | 106 | Evacuees   (newly arrived) | 6  days | 6 days | Random selection |
| **3. Ridenour**  **2007** | Hurricane  U.S.A. | modified (CDC-WVU) | Face to face  (household) | 164 | Evacuees | 12  days | 3 days | Convenience sample |
| **4.Schnitzler 2007** | Flooding GE | new | Telephone  (individual) | 477 | Home   (ex-evacuees) | 3 months | 2 days | Random selection |
| **5. CDC** Jan **2006** | Hurricane USA | unknown | Face to face  (household & individual) | 166 | Home   (ex-evacuees) | 1 month and 19 days | 6 days | Cluster  Sampling |
| **6. CDC** April **2006** | Hurricane USA | translated | Face to face (household)  Paper & PDA | 165 | Home | 8  days | 2 days | random selection |
| **7. CDC**  March **2006 A** | Hurricane USA | unknown | Face to face  (household) | 1.360 | Evacuees | 17 days | 4 days | All heads of households |
| **8.CDC** March **2006** B | Hurricane USA | unknown | Face to face (household) | 197 | Home | 16  days | 2 days | Cluster sampling |
| **9. Bayleyegn  2006** | Hurricane USA | modified (CDC)  &  translated | Face to face  (household)   Paper | 420 | Home | 6 days | 3 days | Modified  cluster  sampling |
| **10. Kamp   2006** | Firework Disaster NL | new  & translated | Self-reported  (individual/ adult)  Paper | 3.792 | Research Center | 3 weeks | 8 days | All survivors  invited |
| **11. Rodriquez 2006** | Hurricane  U.S.A. | modified (CDC) | Face to face  (household) | 197 | Evacuees | 10  days | 2 days | Convenience sample |
| **12. Brennan 2005** | Tsunami Indonesia | modified  (UN) | Face to face (household) | 316 | Home | 16  days | 2 days | Systematic random sample |
| **13. CDC** Sept **2004** | Hurricane USA | unknown | Face to face  (household with an older adult) | 3 assessments (in 3 counties):  n = 198 n = 192  n = 205 | Home | 14  days | D = 5 days:  D = 1 D = 1 D = 3 | Probality-based cluster selection |
| **14. CDC**  Sept **2004** | Hurricane USA | translated | Face to face  (household) | 210 | Home | 3 days | 1 day | Modified cluster sampling |
| **15. Chen et 2003** | Earthquake Taiwan | modified  (CDC) | Face to face  (household) | 2 assessments (in 2 counties)  n = 210  n = 210 | Evacuees | 5 days | 1 day | Modified cluster sampling |
| **16. CDC** May **2002** | Tropical Storm  USA | modified  (CDC) | Face to face  (household) | 420 | Home | 1 week | 1 day | Modified cluster  sampling |
| **17. CDC** Sept **2002** | Terrorist Attack USA | new | Face to face  (household) | 414 | Home | 1 month and 2 weeks | 9 days | Random Selection |
| **18. Daley  2001** | Earthquake Turkey | modified &  translated | Face to face  (household) | 230 | Evacuees | 9  days | 2 days | Modified cluster sampling |
| **19. CDC**  July **1998** | Ice Storm USA | modified | Face to face (household) | 111 | Home | 10 days | 1 day | Modified cluster sampling |
| **20.CDC** Feb **1996 1** | Hurricane USA | unknown | Face tot face (household) | unknown | Home | 1 week & 2 weeks  (survey repeated) | 1 day | Cluster sampling |
| **21. CDC** June **1993** | Hurricane  USA | unknown | Face tot face (household) | 1.353 | Home | 2 months and 10 days | 11 days | Cluster sampling |
| **22. CDC** Sept **1992** | Hurricane USA | unknown | Face to face  (household) | 211 | Home | 3 AND 10 days  (survey repeated) | 5 hours | Cluster sampling |

*Preparation situation**:** 1. modified = a checklist that is modified to the specific disaster situation 2. new = a newly designed checklist 3. translated = translation of an existing checklist.
****** Way of administration: 1. face to face = face to face interview 2. telephone = telephonic interview 3. email = interview send by email 4. self-reported = questionnaire is self-reported.

# Assessment level: 1. individual = individual level 2. household = household level of which the head or representative of the household was interviewed 3. group = group level.
^ Location population during assessment 1. evacuees = in evacuee centres 2. home = in their own homes 3. in a research centre 4. in homes of family and friends.
