# Supplementary material for: Rapid Health and Needs assessments after disasters: a systematic review
Source: BMC Public Health. 2010 Jun 1;10:295. doi: 10.1186/1471-2458-10-295 (PMC2889870; doi:10.1186/1471-2458-10-295)
Supplement: Additional file 2 — Assessments conducted with use of registries. Overview and characteristics of included articles. [file 1471-2458-10-295-S2.DOC]

**Additional file 2. Assessments conducted with use of registries**

| **First Author**  &  Publication Year | **Type of Disaster** Country | **Type of DATA RECORD*** | **Method of**  registration** (assessment level)# | **ACCESS TO DATA** | **N=**  **Participants** | **popu-**  **lation^** | **Time after disastEr** | **Duration** | **assessment theme’s** | | | | | | | |
| --- | --- | --- | --- | --- | --- | --- | --- | --- | --- | --- | --- | --- | --- | --- | --- | --- |
|  |  |  |  |  |  |  |  |  | Demo | | Health Needs | | Health Status | Practical N & S | | |
| **1. Cookson 2008** | Hurricane  USA | SendSS: State Electronic  Notifiable Disease  Surveillance System -->  Input:  -Internet  based   surveillance  form  +  Hospital  registration  - ED records | Electronic      Actively recorded (individual)     Regular registration (individual) | Conducted by: district disease surveillance team (in shelters)  retrospective | 125.000 | Evacuees | 3 days | 3 weeks | X | |  | | X |  | | |
| **2. CDC** July **2008** | Wildfires USA | Biosense ( national  public health  system)  Input: Hospital registration - ED records | Electronic     Regular registration (individual) | monitored by CDC personnel   recorded by medical staff | 5.884 | Visitors hospital ED’s | 1 day | 9 days | X | | |  | X | |  | |
| **3. Brown  2007** | Hurricane USA | Military hospital  Registration:  -Patient records +  -Pharmacy records | Electronic  Regular registration  (individual) | Telephone  request  retrospective | 39.910 | Evacuees | 1  days | 1 month & 3 days | X | | |  | X | |  | |
| **4. Ridenour  2007** | Hurricane USA | Red Cross  household   registration | Regular registration  (household) | unknown | 210 | Evacuees | 11 days | 4 days | X |  | | |  | | | X |
| **5. Jhung   2006** | Hurricane USA | - DMAT*** pharmacy records  - Retail pharmacy records | Actively recorded (individual)  Regular registration  (individual)  Electronic | Care  providers entered data into surveillance system | 95.000 | Evacuees | 4  days | 27 days |  | X | | | X | | |  |
| **6. Sullivent 2006** | Hurricane USA | Hospital registration:  - medical   records  - one-page   form | Regular registration  (individual)  Actively recorded (individual)  🡪 on Paper | Abstracted by CDC personnel  recorded by medical staff | 11.000 | Evacuees & Home 🡪  Hospitalized  - residents  - relief workers | 10  days | 5 weeks | X |  | | | X | | |  |
| **7. CDC** March **2006 C** | Hurricane USA | Medical clinic registration:  - Reports of   syndromes  - medical  records  Hospital registration:  - ED records | Actively recorded (individual)  🡪 on Paper  Actively recorded  (individual)  Regular registration  (individual) | Faxed daily    unknown   ED personnel requested to identify and report evacuee visits to infection control practitioners | 509 | Evacuees | 9  days | 16 days | X |  | | | X | | |  |
| **8. CDC** March **2006 D** | Hurricane  USA | Hospital registration:  - ED records  DMAT’s & medical centres:  - medical  records | Actively recorded (individual)  Actively recorded (individual)  🡪 mostly on paper | surveillance team travelled to ED’s,  DMATs and  centres | 11.424 | Visitors ED’s, DMATs and medical centres | 7 days | 7 days | X |  | | | X | | |  |
| **9. Chen   2003** | Earthquake Taiwan | Temporary Medical Service System registration:  - medical  records  Registration Ministry of Interior | Regular registration  (individual)  Regular registration  (individual) | Reviewed by disaster surveillance  team (deployed to  TMSS’s)  unknown | 70.718 | Evacuees & Home 🡪  Visitors TMSS’s | 13  days | 1,5 month | X |  | | | X | | |  |
| **10. CDC  2002** | Terrorist Attack USA | Hospital  Registration:  - ED records  - medical  records | Regular registration  (individual)  Regular registration  (individual) | unknown | 1.103 | Evacuees & Home 🡪  Visitors hospitals & hospitals ED’s | 6  ours | 2 days | X |  | | | X | | |  |
| **11. Ogden 2001** | Flooding USA | Hospital Registration:  - ED records | Regular registration  (individual) | LOPH requested reports by contacting ED nurses | 25.000 | Evacuees & Home 🡪  Visitors hospital ED’s | 1 week | 15 days |  |  | | | X | | |  |
| **12. CDC** July **1998** | Ice Storm USA | Hospital & Medical Center Registration:  - ED records | Regular registration  (individual) | unknown | 2.586 | Evacuees & Home 🡪  Visitors hospital ED’s  (in the most heavily affected region) | First day | 12 days |  |  | | | X | | |  |
| **13. CDC** Feb **1996 II** | Hurricane USA | In Hospital & Medical Center & DMAT’s   - surveillance  systems | Actively recorded (individual) | unknown | 3.265 | Evacuees & Home 🡪  Visitors Hospital & Medical Center & DMAT’s | 1 day  till 15 days | 15 days |  |  | | | X | | |  |
| **14. CDC** Feb **1996 III** | Hurricane USA | Hospital registration:  - ED records | Regular registration  (individual) | unknown | 1.131 | Evacuees & Home 🡪  Visitors Hospitals | 1  day | 6 days |  |  | | | X | | |  |
| **15. Lee 1993** | Hurricane USA | Free care sites:  - medical  records  Hospital registration:  - ED records | Actively recorded (individual)  Regular registration  (individual) | recorded by medical staff 🡪  data collected +  classified by  epidemio-logists | 59.219 | Evacuees & Home 🡪  Visitors Hospitals ED’s &  Free care  sites | 1 day | 1 month |  |  | | | X | | |  |
| **16. CDC** April **1993** | Hurricane USA | In Hospital ER:  - Surveillance  system | Actively recorded (individual)  🡪 Paper | Telephone calls to  ER personnel | 462 | Evacuees & Home 🡪  Visitors ER Hospitals | 2  days  pre-disaster | 1 month | X |  | | | X | | |  |

***** Type of data record **=** for example general practitioner registration or hospital registration system

****** Way of registration: 1. regular registration = data abstracted from a regular existing registration system 2. actively recorded = data actively recorded on a structured form specific designed
 for a post-disaster situation.
# Assessment level: individual = individual level 2. household = household level of which the head or representative of the household was interviewed 4. group = group level.

^ Location population during assessment 1. evacuees = in evacuee centres 2. home = in their own homes 3. in a research centre 4. in homes of family and friends.

*** DMAT = Disaster Medical Assistance Team: a group of medical professionals who can provide medical care during a disaster or other event.
